# A Robust Hybrid Weighting Scheme Based on IQRBOW and Entropy for MCDM: Stability and Advantage Criteria in the VIKOR Framework

**DOI:** 10.3390/e27080867

**Published:** 2025-08-15

**Authors:** Ali Erbey, Üzeyir Fidan, Cemil Gündüz

**Affiliations:** Department of Computer Programming, Distance Education Vocational School, Usak University, Usak 64200, Türkiye; uzeyir.fidan@usak.edu.tr (Ü.F.); cemil.gunduz@usak.edu.tr (C.G.)

**Keywords:** decision support systems, multi-criteria decision-making, interquartile range, entropy weighting, hybrid weighting method, outlier robustness

## Abstract

In multi-criteria decision-making (MCDM) environments characterized by uncertainty and data irregularities, the reliability of weighting methods becomes critical for ensuring robust and accurate decisions. This study introduces a novel hybrid objective weighting method—IQRBOW-E (Interquartile Range-Based Objective Weighting with Entropy)—which dynamically combines the statistical robustness of the IQRBOW method with the information sensitivity of Entropy through a tunable parameter β. The method allows decision-makers to flexibly control the trade-off between robustness and information contribution, enhancing the adaptability of decision support systems. A comprehensive experimental design involving ten simulation scenarios was implemented, in which the number of criteria, alternatives, and outlier ratios were varied. The IQRBOW-E method was integrated into the VIKOR framework and evaluated through average Q values, stability ratios, SRD scores, and the Friedman test. The results indicate that the proposed hybrid approach achieves superior decision stability and performance, particularly in data environments with increasing outlier contamination. Optimal β values were shown to shift systematically depending on data conditions, highlighting the model’s sensitivity and adaptability. This study not only advances the methodological landscape of MCDM by introducing a parameterized hybrid weighting model but also contributes a robust and generalizable weighting infrastructure for modern decision-making under uncertainty.

## 1. Introduction

In today’s complex decision-making environments, the combination of uncertainty, information overload, and the need to evaluate multiple criteria has made it more important than ever for decision-makers to have access to supportive tools. In particular, Decision Support Systems (DSSs) provide analytical support in such uncertain conditions, helping to create decision-making processes and enabling organizational strategies to be grounded on a more solid foundation [1,2]. However, the success of these systems to a large extent depends on the accuracy, reliability, and robustness of the weighting methods integrated into the decision-making process [3,4]. In the literature, weighting methods are generally classified into three main categories: subjective, objective, and hybrid approaches. Subjective methods rely on expert judgments or survey-based preferences, whereas objective methods are based on data-driven statistical and mathematical computations. Hybrid methods, on the other hand, generally aim to combine the strengths of both subjective and objective approaches, offering more flexible and comprehensive solutions for complex decision-making problems. The increasing importance of hybrid approaches has been emphasized in various domains, especially in environments where decision ambiguity coexists with data irregularity [5,6,7].

In this context, multi-criteria decision-making (MCDM) methods have gained significant importance in both the academic literature and real-world applications. In MCDM processes, objectively determining the relative importance levels of criteria is a fundamental step that directly affects the integrity of the decision. Although many weighting methods have been proposed in the literature for this purpose, each has its own limitations. For example, the Entropy method, which is an information-based approach, measures differences between criteria through statistical diversity but is sensitive to outliers in the data [8,9]. On the other hand, the Interquartile Range-Based Objective Weighting (IQRBOW) method, which utilizes the distributional characteristics of the data, produces more robust results, especially in data sets containing outliers [10]. However, this method may be limited in terms of information content sensitivity.

In this study, a new hybrid structure called IQR-Based Objective Weighting with Entropy (IQRBOW-E) is introduced, which combines the strengths of the two aforementioned methods. IQRBOW-E enables decision-makers to establish a flexible balance between statistical robustness and information sensitivity through the β parameter. Thus, both the robustness of IQRBOW against outliers and the information-based sensitivity of Entropy are blended together, resulting in a more balanced, flexible, and reliable weighting approach.

The proposed IQRBOW-E method is not limited to MCDM applications and can also be integrated into the core data processing infrastructure of DSSs. The robustness of the method has been tested under various scenarios and analyzed in detail in terms of advantages and stability conditions. Additionally, this study aims to demonstrate that hybrid weighting can maintain stability in changing data structures, thereby providing a reliable foundation for decision support mechanisms.

The main contributions of this study are summarized as follows:

- A novel hybrid objective weighting method (IQRBOW-E) is proposed, combining IQR-based robustness and Entropy-based information sensitivity.
- The method provides a flexible structure for adjusting the trade-off between robustness and information contribution in weighting processes through a tunable parameter β.
- An extensive simulation-based experimental design with varying numbers of criteria, alternatives, and outlier ratios is developed to evaluate performance.
- The IQRBOW-E method is integrated into the VIKOR framework and evaluated using statistical and decision-theoretic criteria such as Q values, SRD scores, advantage/stability ratios, and the Friedman test.
- The findings demonstrate that IQRBOW-E outperforms traditional methods, especially in environments with high uncertainty and outlier contamination.

The remainder of this paper is organized as follows: Section 2 presents a review of related works on weighting methods in MCDM. Section 3 introduces the methodology, including the development of the IQRBOW-E method and the experimental design. Section 4 provides the simulation results and statistical analyses under various decision scenarios. Section 5 discusses the implications of the findings. Finally, Section 6 concludes the paper and outlines directions for future research.

## 2. Related Works

In MCDM processes, the accurate determination of the relative importance levels of decision criteria is considered one of the key determinants of final decision quality. In this context, weighting procedures are examined in the literature under two main groups: subjective and objective weighting methods [10]. Subjective methods are generally based on decision-makers’ judgments, expert opinions, or survey-based evaluations and heavily incorporate the influence of human factors [11]. In contrast, objective methods adopt a systematic, repeatable, and computation-based approach based on numerical data in the decision matrix [10]. Prominent examples of objective methods include Entropy [9], IQRBOW [10], Standard Deviation [12], CRiteria Importance Through Intercriteria Correlation (CRITIC) [13], MEthod based on the Removal Effects of Criteria (MEREC) [14], Gini Index [15], and PCA-based [16] weighting methods. Each of these methods is designed to assign a priority level to criteria based on their data distribution or information contribution. However, most of these methods are either susceptible to outliers due to their focus on variance alone or, even if they are information theory-based, fail to tolerate irregularities in the data structure. These challenges can limit the decision support capacity of objective methods under conditions where real-world data contain uncertainty, incompleteness, and noise.

One of the most widely used methods among these is Entropy-based weighting, which is based on Shannon’s approach, the founder of information theory. It assigns greater weight to criteria with high information content by calculating the uncertainty of criterion values [9]. The Entropy method is successful in highlighting criteria with high discriminatory power by considering the internal variations of the criteria in the decision matrix. Indeed, in this regard, it produces very effective results, especially in consistent and balanced data sets. However, the method is sensitive to outliers in the data, as probability-based approaches normalized according to the total of criterion values can lead to significant weight shifts even with minor data deviations. Additionally, the Entropy method is limited in terms of statistical robustness because it focuses on the distribution density rather than the range of criterion values.

As an alternative, the IQRBOW method proposed in the recent literature evaluates the importance level of criteria based on the interquartile range (IQR), a statistical dispersion measure [10]. The IQR represents the middle 50% of the data set, which is calculated as the difference between the first and third quartiles ($Q_{3}-Q_{1}$), and is resistant to outliers. In this regard, IQRBOW allows criterion weights to be obtained without distortion, especially in decision-making environments containing outliers. Compared to Entropy, IQRBOW offers a more robust approach and can produce reliable results regardless of the symmetry or variance of the data set. However, it has low sensitivity to the information density in the data, as it only considers the distributional width and does not take into account the discriminative information provided by the criterion. This can cause IQRBOW to be ineffective in some decision-making environments with information deficiencies or homogeneous data sets.

In recent years, hybrid weighting methods have been developed to combine the advantages of different structures, such as Entropy and IQRBOW [17]. Some of these studies combine weights through arithmetic averages or weighted combinations [11], while others prefer approaches such as fuzzy logic [18,19], the maximum Entropy principle, or ideal solution reference evaluation [20,21]. However, most of these hybrid methods neither offer an adjustable parameter (e.g., β) to balance the contribution of the two methodologies nor provide a systematic approach to optimize such a parameter. This prevents decision-makers from establishing a flexible balance according to the data structure, thereby limiting the potential of the hybrid structure.

The IQRBOW-E method, developed to address these shortcomings, is an innovative weighting approach that combines statistical robustness with information sensitivity. The method integrates IQRBOW’s robust structure against outliers and Entropy’s information theory-based discriminative power through the β parameter in a dynamic manner. As a result, decision-makers or system designers can control which method is dominant based on the structure of the data set and develop an adaptive solution strategy instead of a fixed weighting structure. IQRBOW-E can be evaluated not only as a decision-making algorithm but also as a flexible and robust infrastructure that can be integrated into decision support systems.

## 3. Methodology

The IQRBOW-E method proposed in this study is a new objective weighting approach that aims to calculate criterion weights in multi-criteria decision-making processes based on both information sensitivity and statistical robustness. The method combines two fundamental weighting systems—Entropy and IQRBOW—to offer a hybrid system based on the β parameter, which establishes a dynamic balance between these methods. Unlike fixed weight combinations, this hybrid approach provides an adaptable framework tailored to the nature of the data set and is designed for integration with decision support systems.

### 3.1. Data Set Creation and Normalization

The first and fundamental step in the decision-making process is to clearly define the alternatives and evaluation criteria. The decision matrix used in this study is modeled in a structure that includes alternatives in rows and criteria in columns. Each cell represents the numerical value that a specific alternative receives in terms of a specific criterion.

Some of the criteria in the decision matrix are of the “benefit” type, meaning that higher values are preferred. Others are of the “cost” type, where lower values are preferred. To compare these two different types of criteria under the same framework, the criterion values must be scaled (normalized) using an appropriate method.

In this study, the min–max normalization method was used to eliminate biases that may arise from criteria having different scales and units (Equations (1) and (2)).

(1) $$x_{ij\;\left(benefit\right)}^{\prime }=\frac{x_{ij}-min\left(x_{j}\right)}{\max\left(x_{j}\right)-min\left(x_{j}\right)}$$

(2) $$x_{ij\;\left(cost\right)}^{\prime }=\frac{max\left(x_{j}\right)-x_{ij}}{\max\left(x_{j}\right)-min\left(x_{j}\right)}$$

Here, $x_{ij}^{\prime }$ represents the normalized value of the alternative i and criterion j; $x_{ij}$ represents the original criterion value; $min\left(x_{j}\right)$ and $\max\left(x_{j}\right)$ represents the minimum and maximum values of the jth criterion, respectively.

In this method, the values of each criterion are converted to a range between 0 and 1 according to their own minimum and maximum ranges. For benefit-type criteria are scaled so that lower values tend toward 0 and higher values tend toward 1, whereas cost-type criteria follow the opposite trend. This ensures that all criteria are assessed on a unified scale.

Min–max normalization enables direct comparison between alternatives without distorting the information content of the decision matrix. It also facilitates the statistical soundness of subsequent weighting and ranking processes.

### 3.2. Calculation of Entropy Weights

The Entropy method is an objective weighting approach that evaluates the contribution of each criterion to the decision process using an information theory-based measurement. In this method, the greater the diversity of criterion values, the higher the information content of that criterion is considered to be. Accordingly, criteria with low diversity are assumed to contribute less to the decision process and are assigned lower weights.

When calculating Entropy weights, each normalized criterion cell value is first divided by its column total to obtain a probability distribution (Equation (3)):

(3) $$p_{ij}=\frac{x_{ij}^{\prime }}{\sum_{i=1}^{m}x_{ij}^{\prime }}$$

Here, $p_{ij}$ represents the normalized and standardized (probability) value of alternative i for criterion j; $x_{ij}^{\prime }$ represents the value normalized using the min–max method; and m is the total number of alternatives.

Prior to computing Equation (3), each raw element $x_{ij}^{\prime }$ is scaled to the [0, 1] interval by the monotonic min–max operator in Equation (2). This preprocessing step (i) converts cost-type criteria to a common benefit direction, (ii) ensures all values are strictly non-negative so that $p_{ij}\cdot \ln p_{ij}$ is well-defined, and (iii) prevents large-magnitude attributes from numerically dominating the Entropy term.

The Entropy value of each criterion is calculated using the information theory approach with the following formula (Equation (4)):

(4) $$e_{j}=-k\cdot \sum_{i=1}^{m}p_{ij}\cdot \ln p_{ij}$$

Here, $e_{j}$ represents the Entropy value of criterion j; while $k=\frac{1}{ln\left(m\right)}$ serves as a normalization factor (fixed for a given decision matrix). This factor rescales $e_{j}$ so that $0\;\leq \;e_{j}\;\leq \;1$ and its dependence on the number of alternatives m guarantees comparability across different matrix sizes. As the Entropy value increases, the information value of the criterion decreases. Therefore, the weights are calculated using dj=1−ej, which is the complement of Entropy. The Entropy-based weight of each criterion is obtained as follows (Equation (5)):

(5) $$w_{j}=\frac{dj}{\sum_{j=1}^{n}dj}\;$$

Here, $w_{j}$ is the final Entropy weight of criterion j and n denotes the total number of criteria [9].

### 3.3. Calculation of IQRBOW Weights

IQRBOW is an objective method that uses IQR, a statistical distribution measure, to determine criterion weights [10]. This approach was developed to make the decision-making process more reliable, especially in data sets with outliers. IQR represents the middle 50% of the data and provides a robust measure of variability that is not affected by extreme values. Unlike standard-deviation- or variance-based weighting schemes, IQRBOW measures dispersion with the inter-quartile range (IQR)—a statistic that remains unaffected until at least 25% of the observations are contaminated with outliers. Because variance increases quadratically with extreme values, even a single outlier can dominate weights when variance is used, whereas the IQR changes only if the quartiles themselves shift.

In the IQRBOW method, the importance level of the criteria is calculated in direct proportion to the spread of each criterion in the data distribution. Criteria with higher IQR values are assumed to have greater variability and contribute more to the decision-making process.

For each criterion, the IQR value is calculated as the difference between the third quartile ($Q_{3}$) and the first quartile ($Q_{1}$) (Equation (6)):

(6) $$IQR_{j}=Q_{3}-Q_{1}$$

Here, $IQR_{j}$ represents the IQR value for criterion j, while $Q_{3}$ and $Q_{1}$ represent the third and first quartile values for that criterion, respectively.

Since IQR measures the spread of values concentrated around the center of the data, it is highly insensitive to outliers. This feature enhances the statistical robustness of the method in decision-making processes.

After calculating the IQR values for all criteria, these values are normalized by dividing them by the total IQR, and the criterion weights are calculated as follows (Equation (7)):

(7) $$w_{j}=\frac{IQRj}{\sum_{j=1}^{n}IQRj}$$

The IQRBOW method is resistant to outliers because it focuses on the central part of the statistical distribution, producing reliable results, especially when the data set deviates from normality. Instead of evaluating criteria using statistical measures such as mean and variance, which are sensitive to outliers, evaluating them using a more robust structure ensures the consistency of decision quality. With this feature, IQRBOW offers higher stability and reliability compared to traditional weighting methods [10].

### 3.4. Calculation of IQRBOW-E Weights

IQRBOW-E is a flexible and controllable hybrid weighting approach developed by combining the strengths of the Entropy and IQRBOW methods. The main objective of this method is to integrate the statistical robustness offered by the IQRBOW method into the decision-making process while maintaining the information-based sensitivity obtained by the Entropy method.

The IQRBOW-E method is defined by the β parameter, which enables the decision-maker to balance the effect of the two methods. It is defined in the range $\beta \;\in \;\left[0,\;1\right]$ and controls the balance in the hybrid weight structure. Thanks to this parameter, the decision-maker can choose between an information-sensitive or robustness-focused approach depending on the data structure. The method’s computational process is carried out in a single step (Equation (8)):

(8) $$w_{j}^{IQRBOW-E}=\beta \cdot \;w_{j}^{IQRBOW}+\left(1-\beta \right)\cdot \;w_{j}^{Entropy}$$

In this formula, $w_{j}^{IQRBOW}$ represents the weight of criterion j computed using the IQRBOW method (see Equation (7)), and $w_{j}^{Entropy}$ denotes the weight calculated using the Entropy method (see Equation (5)). The parameter $\beta \in \left[0,1\right]$ is used to balance the influence of the two methods, allowing for a flexible integration of robustness and information sensitivity. This formula processes both weight vectors in a normalized form of the same size and creates a vector that is scaled to the $\left[0,1\right]$ range and normalized to be the sum of the resulting hybrid weights is 1.

- When β = 1 is true, the method is reduced to pure IQRBOW, i.e., it is based entirely on statistical robustness.
- When β = 0 is true, the method becomes pure Entropy, creating a structure based on information diversity.
- In the 0 < β < 1 range, a controlled blend of IQRBOW and Entropy is obtained.

Thanks to this structure, IQRBOW-E offers an adaptable weighting system for different data structures and decision problems. For example, if the data set contains outliers, a higher β value is preferred, while lower β values are recommended when information differences between criteria are prominent. This flexibility makes the method both theoretically robust and dynamically applicable within decision support systems. Additionally, this structure can be directly integrated not only with ranking-focused methods like VIse Kriterijumska Optimizacija I Kompromisno Resenje (VIKOR) [22] but also with Technique for Order of Preference by Similarity to Ideal Solution (TOPSIS) [8], Evaluation based on Distance from Average Solution (EDAS) [23], Measurement of Alternatives and Ranking according to COmpromise Solution (MARCOS) [24], Grey Relational Analysis [25], and other objective decision models. Thus, IQRBOW-E provides a modular, robust, and general-purpose weighting infrastructure for both classical and modern MCDM applications.

### 3.5. Experimental Design and Application Scenarios

**Figure 1:**
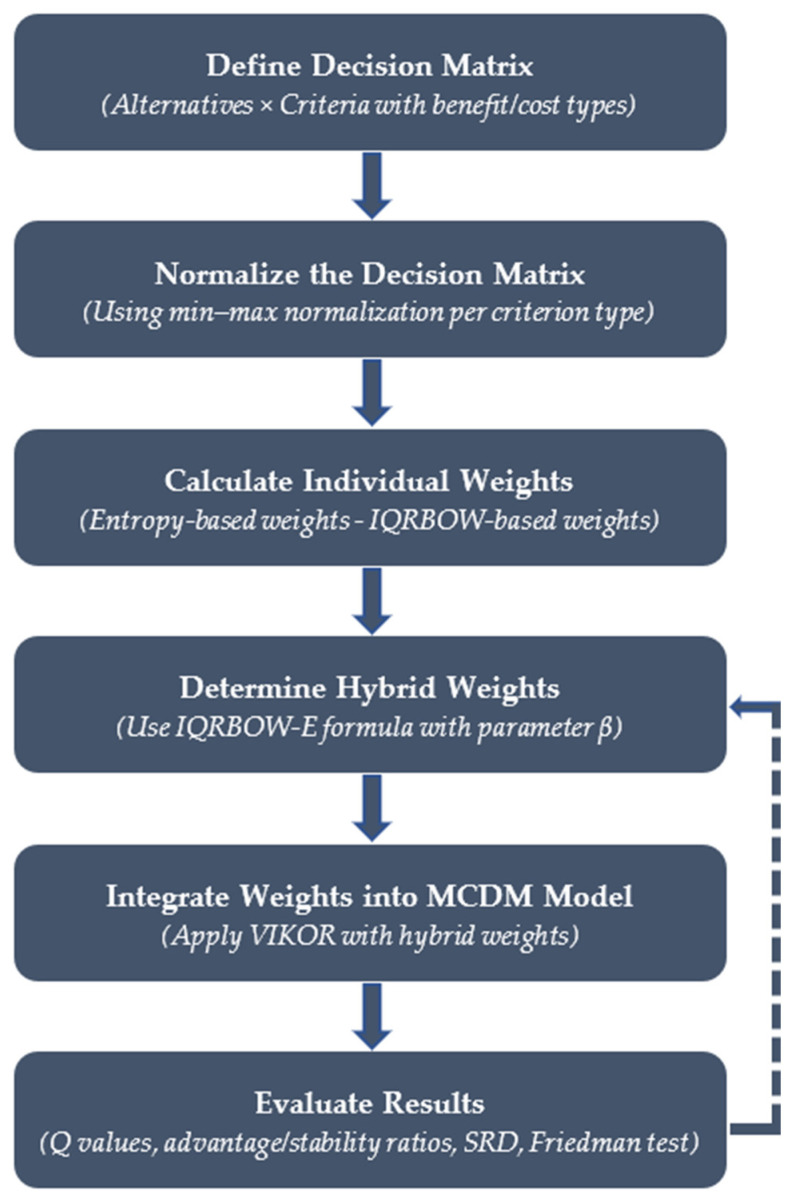
Flowchart of the proposed IQRBOW-E methodology.

The overall procedure of the proposed IQRBOW-E method is summarized in a flowchart (Figure 1), which visualizes the key steps from synthetic data generation to decision evaluation and highlights the feedback mechanism that enables recalibration of hybrid weights via the β parameter when needed.

Figure 1, illustrating the workflow from data generation and normalization to hybrid weighting, integration into VIKOR, and result evaluation. A feedback loop from the evaluation step to hybrid weight recalibration is also included to reflect the model’s adaptability through β tuning.

The proposed IQRBOW-E method has been tested under various experimental scenarios to understand the effects of differences in data structure on weighting results and to evaluate the robustness of the method. In this context, the experimental design is based on controlled simulations aimed at observing the effects of different numbers of criteria and alternatives, outlier ratios, and the β parameter in the hybrid structure.

To evaluate the impact of the proposed IQRBOW-E method on decision-making outcomes, the VIKOR method was selected as the ranking framework. VIKOR is particularly suitable for decision problems characterized by conflicting criteria and uncertain data, which aligns with the objectives of our study. Unlike other MCDM methods, VIKOR incorporates two key decision principles—acceptable advantage and acceptable stability—which make it well-suited for assessing both performance and robustness under varying data structures. Consistent with VIKOR’s original formulation, we regard these principles as satisfied only when (i) the acceptable-advantage gap exceeds its theoretical threshold, i.e., ΔQ ≥ 1/(m − 1), and (ii) the same alternative simultaneously attains first place in either the S or R ranking, yielding a 100% stability ratio. In VIKOR, S (group utility) is the weighted sum of normalized deviations from the positive-ideal across all criteria (lower S indicates better overall performance), whereas R (individual regret) is the maximum of those weighted deviations on any single criterion (capturing the worst-case shortfall) [22]. These features enabled us to systematically evaluate how different β values influence not only ranking outcomes but also consistency and decision reliability.

In each experimental condition, decision matrices were synthetically generated, and criterion weights were calculated using IQRBOW-E. The synthetic data were generated using a simulation-based approach with controlled randomness. Each criterion in the decision matrix was produced using independent normal distributions whose means were randomly drawn from a uniform range between 50 and 150, and standard deviations were randomly selected between 5 and 20. These ranges were selected to reflect a moderate level of variability commonly observed in real-world MCDM applications. They help ensure that the data contain sufficient dispersion without being unrealistically volatile or overly uniform. This setting allows for meaningful normalization and reliable interpretation of weighting behaviors. Additionally, outliers were introduced in relevant scenarios by replacing selected values with extreme values calculated based on the upper bound of the interquartile range (IQR). The random generation process was seeded (Seed = 42) to ensure reproducibility across all simulations, which were repeated 1000 times for each scenario. These weights were then evaluated in terms of ranking performance using the VIKOR method, one of the commonly used methods in multi-criteria decision-making. The objective here is to identify under which conditions IQRBOW-E produces the most stable, advantageous, and reliable decisions.

The grid-search routine is not tied to the VIKOR-specific quantity Q. When another ranking model is employed, the primary ordering statistic of that model can simply replace Q in the algorithm. For instance, in TOPSIS, one may minimize the average closeness coefficient $\bar{CC}$ (distance to the positive ideal solution) while retaining the same ranking–stability and advantage checks. Likewise, for PROMETHEE, the net outranking flow $\phi_{i}$ could serve as the optimization objective. In this way, the β selected always represents the point at which the hybrid weighting yields the most favorable performance for the target MCDM algorithm.

Within the scope of the experimental study, a series of controlled parameters were used to evaluate the performance of the proposed IQRBOW-E method in different data structures. These parameters are components that determine the basic structure of the decision matrix and the experimental conditions [26]. First, two basic structural variables, namely, the number of criteria and the number of alternatives, were considered. These variables determine the size and complexity of the decision matrix, enabling the method’s stability to be tested at different scales. Second, the outlier ratio was varied to measure the statistical robustness-based advantage of IQRBOW-E. In this context, outliers were systematically added to the data set at rates of 0% (no outliers), 1%, 5%, and 10% [27]. In this study, the outlier ratio is defined as the proportion of decision matrix elements that are intentionally replaced by extreme values. The β value, the most fundamental control parameter of the hybrid structure, was scanned with increasing values between 0 and 1, and the optimal equilibrium point was determined for each scenario. In this context, the optimal equilibrium point refers to the value β that simultaneously (i) minimizes the average VIKOR compromise measure Q, (ii) satisfies VIKOR’s advantage and stability conditions ($\Delta Q\;\geq \;1⁄\left(m\;-\;1\right)\;and\;S\;=\;100\%$), and (iii) lies within the largest contiguous β-interval fulfilling both criteria. Additionally, to obtain reliable and generalizable results under all conditions, each scenario was repeated 1000 times, and a fixed random number generator (seed) was used to eliminate the effect of randomness [28,29]. Thanks to this multi-dimensional parameter set, both the knowledge-based and robustness-based properties of IQRBOW-E were thoroughly tested, and the method’s applicability to different decision problems was scientifically demonstrated. The scenarios are summarized in Table 1.

The IQRBOW-E method was integrated into the VIKOR method to measure the impact of weights on the decision-making process. VIKOR evaluates alternatives based on both group benefit (S) and maximum dissatisfaction (R) criteria and ranks them using the Q value [30]. The determination of the best alternative was analyzed not only based on the Q value but also through VIKOR’s two fundamental decision rules: “acceptable advantage” and “acceptable stability” conditions [31]. Thus, not only numerical superiority but also compliance with decision theory was tested [22].

Additionally, the average and distribution of Q values (box plots), ranking stability (Sum of Ranking Differences—SRD scores), and variance analysis (Friedman test) were measured under different β values to statistically determine at which β levels IQRBOW-E offers the most robust performance. The effectiveness of the proposed method was assessed using three key evaluation metrics: the stability ratio, the SRD, and the Friedman test, each of which is presented below in formal mathematical notation.

The stability ratio [22] metric measures how often the same alternative ranks first across simulation runs. It is calculated as Equation (9):

(9) $$SR=\frac{n_{stable}}{n_{total}}$$

where $n_{stable}$ is the number of simulations where the top-ranked alternative remains the same, and $n_{total}$ is the total number of simulation iterations (e.g., 1000).

SRD quantifies the deviation of the rankings from an ideal reference (e.g., average ranking). For each alternative $A_{i}$, its SRD score [10,32] is computed as Equation (10):

(10) $$SRD_{i}=\sum_{j=1}^{m}\left|r_{ij}-r_{j}^{ref}\right|$$

where $r_{ij}$ is the rank of alternative $A_{i}$ in simulation j, and $r_{j}^{ref}$ is the reference rank (typically the average rank across simulations for position j). A lower $SRD_{i}$ indicates greater similarity to the ideal ranking.

The Friedman test [33] assesses whether rankings across different β values differ significantly. The test statistic is calculated as Equation (11):

(11) $$X_{F}^{2}=\frac{12}{nk\left(k+1\right)}\sum_{j=1}^{k}R_{j}^{2}-3n\left(k+1\right)$$

where n is the number of alternatives, k is the number of β configurations, and $R_{j}$ is the sum of ranks for the j−th configuration. A p-value below 0.05 indicates significant differences among rankings.

## 4. Results

In this section, the performance of the weights obtained using the proposed IQRBOW-E method under different scenarios has been evaluated through systematic simulations and statistical analyses. In each scenario, variables such as the number of criteria applied to the decision matrix, the number of alternatives, and the outlier ratio were considered; the β parameter was scanned in equal steps between 0 and 1 to analyze the performance dynamics of the hybrid method. The VIKOR method was used as a reference during the evaluation process, and the best alternative was determined for each β based on the obtained Q values. Additionally, the ranking results were examined not only based on the Q values but also according to the fundamental decision criteria of VIKOR, namely the acceptable advantage and acceptable stability criteria. Each simulation output was analyzed in terms of the average Q value, the stability of the best alternative, SRD scores, distribution stability via box plots, and statistical significance via the Friedman test. Thus, it has been demonstrated at which β levels IQRBOW-E most effectively combines its information sensitivity (Entropy) and statistical robustness (IQRBOW) properties.

**Figure 2:**
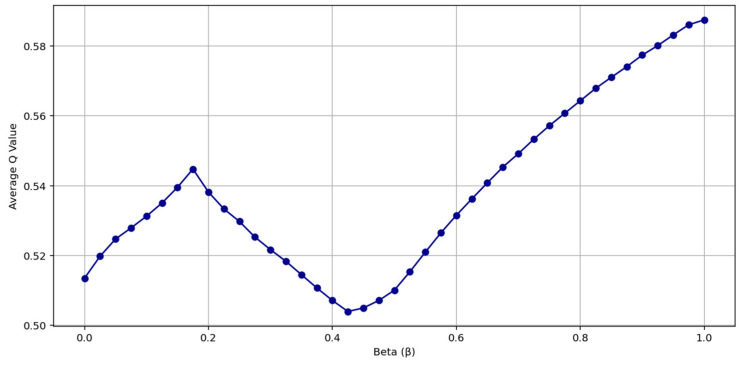
Average Q Value Based on β Value.

The scenario SC1 was considered as the baseline structure representing the base evaluation environment without outliers. In this scenario, the weights obtained using the IQRBOW-E method were tested by changing the β parameter in equal steps between 0 and 1, and the average Q value was calculated for each β. As shown in Figure 2, the average Q value starts at a high level, reaches a minimum around β ≈ 0.43, and then increases again. This indicates that the hybrid structure significantly affects decision quality and that optimizing the β value is necessary.

**Figure 3:**
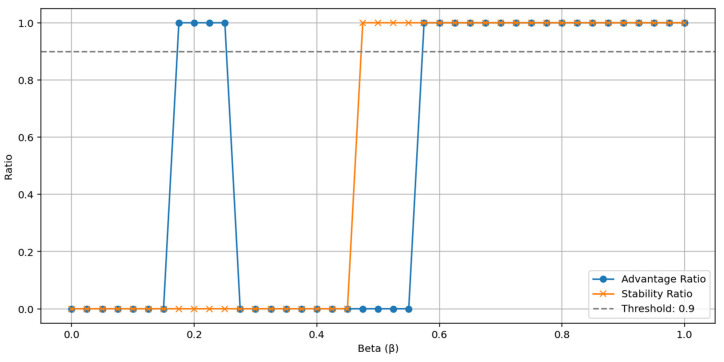
Advantage and Stability Ratios Based on β.

Additionally, the decision rules of the VIKOR method, namely the acceptable advantage and acceptable stability ratios, have also been analyzed. Figure 3 shows the fulfillment rates of these two criteria according to the β value. The range where both advantage and stability conditions are met is concentrated around β ≈ 0.43−0.45; this supports the selection of an optimal β value not only based on the average Q but also according to the decision rules.

Within this scenario, enhanced optimal beta has been determined as β = 0.43, which has both a low average Q value and an advantage and stability ratio of over 90%. Additionally, the absolute optimal β value, which yields the lowest average Q value, has been calculated as 0.425. These values indicate that the decision-maker can achieve both quality and decision reliability within a flexible range.

**Figure 4:**
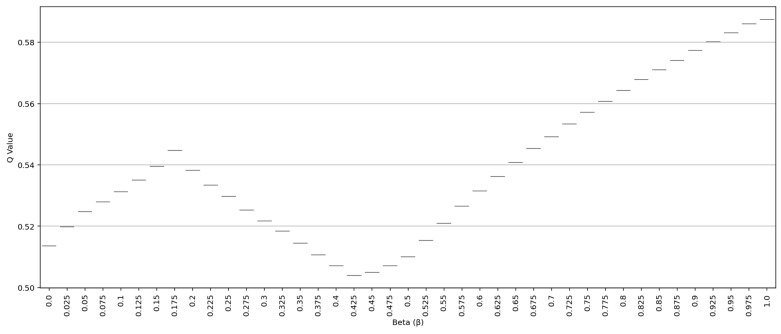
Q Value Distribution According to β Value.

The distribution of Q values is presented in Figure 4 using a box plot; it was observed that within the β = 0.425−0.45 range, not only a low average but also a narrow variance and stable distribution was obtained. Evaluating decision quality with a single value and through its distribution highlights the robust decision-making capability of IQRBOW-E.

**Figure 5:**
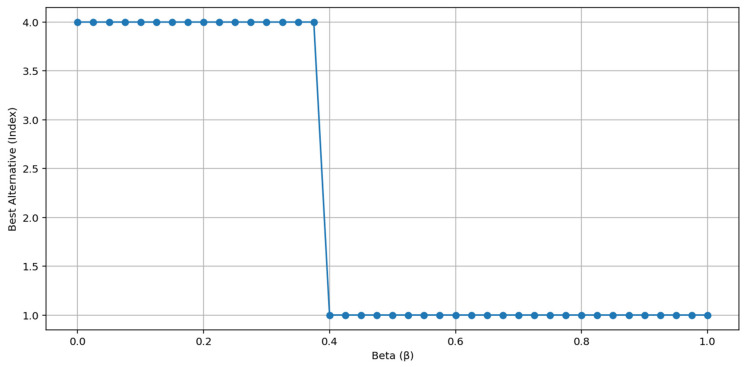
Change in the Best Alternative with Beta Value.

Additionally, the change behavior of the best alternative at different β values is shown in Figure 5. Alternative 1 remains the best alternative throughout the entire scenario until β ≈ 0.60, after which Alternative 4 takes the lead. This observation demonstrates that the hybrid structure offers decision stability within a certain range and that shifts in the weight structure have a limited effect on decisions.

**Figure 6:**
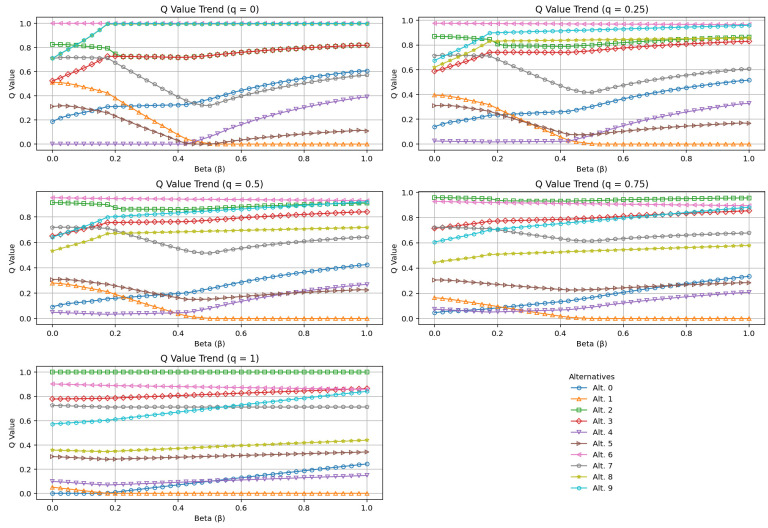
Alternative-Based Q Trends According to Different q Values.

The trends of the Q values of the alternatives according to different q values are presented in five separate sub-panels in Figure 6. This visual demonstrates that the IQRBOW-E method is stable not only with respect to the β parameter but also with respect to the q parameter. In particular, the dominance of Alternative 1 at q = 0.5 and q = 0.75 values indicate that it contributes to the stability of the method.

**Figure 7:**
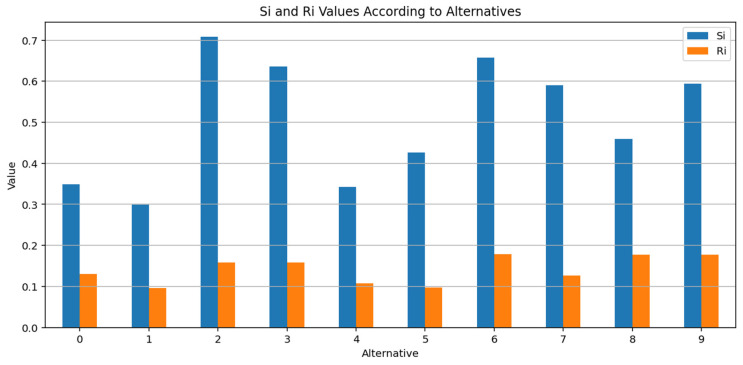
Si and Ri Values According to Alternatives.

The Si and Ri values of the VIKOR components of the decision matrix are presented comparatively in Figure 7. Alternative 1 stands out systematically in terms of Q value thanks to both its low S value (high group benefit) and low R value (low maximum dissatisfaction).

**Figure 8:**
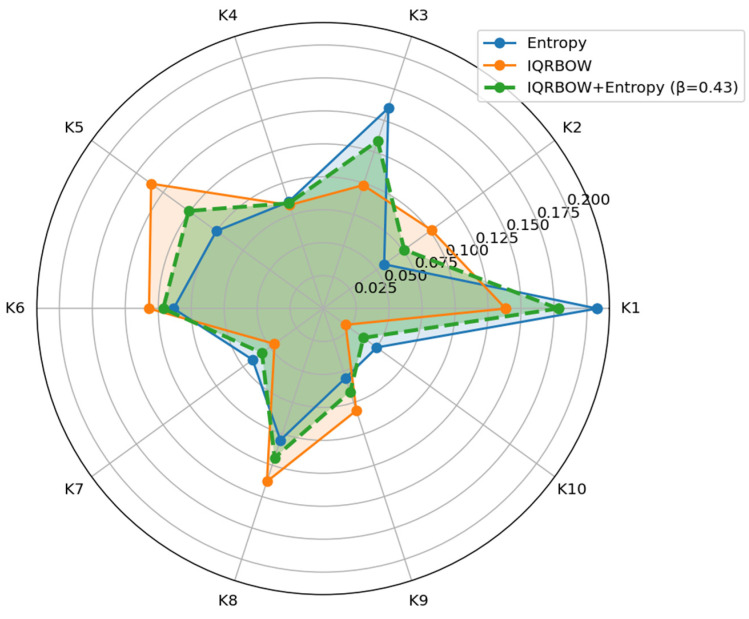
Comparison of Criteria Weights.

On the other hand, Entropy, IQRBOW, and hybrid weights are compared with a radar chart in Figure 8. It can be visually observed how the hybrid structure balances the weights at β = 0.43, while maintaining the robustness effect in criteria where IQRBOW is dominant and the information contribution based on Entropy is effective, especially in criteria such as K1 and K3. Here, K1–K10 are shorthand labels for ten distinct evaluation criteria defined in Table 1; some of these criteria are cost-type and others are benefit-type, as specified in the corresponding scenarios.

**Figure 9:**
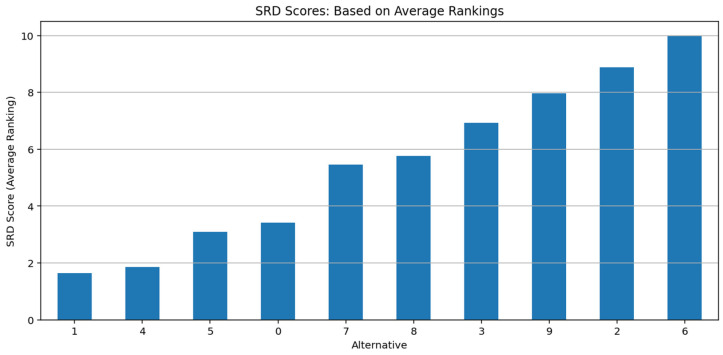
SRD Scores: Based on Average Rankings.

The stability of decision rankings was measured using SRD scores. SRD is an important measure of decision consistency because it is based on average rankings. As shown in Figure 9, Alternative 1 has the lowest SRD value and is the most stable alternative.

Finally, the Friedman test was used to determine the statistical difference between the rankings, and the results were significant ($\chi^{2}\;=\;350.0075,\;p\;=\;0.0000$). This indicates that different β values significantly affect the ranking and that the selection of the optimal β is not only theoretically but also statistically justified.

The impact of the weights obtained using the proposed IQRBOW-E method under various data sets on the decision-making process was evaluated through ten different experimental scenarios. The scenarios included increases in the number of criteria and alternatives, outlier ratios, and combinations of these factors (see Table 2). For each scenario, the β parameter was scanned in equal steps between 0 and 1, and the optimal β was determined based on the obtained Q values. Additionally, decision rules within the VIKOR method, such as acceptable advantage and stability ratios, were also included in the analysis process.

According to the analysis results, it was observed that the optimal β value generally concentrated in the range of 0.4–0.8 in scenarios without outliers (Sc1 and Sc5–Sc9) and low-scale scenarios (10 criteria, 10 alternatives, etc.). This range adequately represents both information-based sensitivity and distributional robustness. When the outlier ratio exceeds 5% (Sc3, Sc4, and Sc10), the optimal β value was observed to increase steadily, highlighting the effect of the IQRBOW-E method. This confirms that IQRBOW-E offers a data-sensitive and adaptive structure.

β optimization, decision stability, and ranking statistics for all scenarios are summarized in Table 2. As the outlier ratio increases, the optimal β value systematically rises, and SRD scores generally become more volatile.

When all scenarios are considered, it was observed that the optimal β value obtained using the IQRBOW-E method not only reduced the average Q scores but also increased ranking stability. The best alternative’s protection ratio remained high, especially in the β = 0.43−0.80 range; the results with the lowest variance in terms of SRD scores were also obtained in this range. Friedman test results revealed significant differences across all scenarios (p < 0.001), indicating that the β parameter statistically influences ranking. These findings suggest that the optimal β value should be determined systematically and based on the data structure, rather than randomly.

## 5. Discussion

This study has demonstrated that the IQRBOW-E method developed for use in decision support systems is not merely a theoretical proposal, but also capable of producing highly flexible, robust, and relevant outputs under varying data sets. The findings clearly demonstrate that hybrid approaches with parametric controllability offer much more reliable decision-making processes than classical single-source weighting methods. In particular, the balance achieved between information sensitivity (Entropy) and statistical robustness (IQRBOW) through the β parameter can be evaluated not only as a mathematical optimization element within the method but also as a flexibility tool capable of modeling the decision-maker’s strategic preferences.

In practical applications, the choice between IQRBOW, Entropy, or their hybrid form (IQRBOW-E) should depend on the characteristics of the data and decision environment. The IQRBOW method is advantageous in scenarios where robustness against outliers and noise is crucial. On the other hand, the Entropy method is more appropriate when discriminating information among criteria is prioritized in a clean, structured data set. The hybrid weighting model (Equation (8)) enables decision-makers to dynamically adjust this trade-off via the β parameter. For instance, higher β values (e.g., β ≥ 0.7) are recommended in volatile environments, whereas lower β values may suit contexts emphasizing informational clarity.

One of the most striking findings worthy of discussion is that, even at low outlier rates, the optimal β value converges toward 1.0 in some scenarios. This indicates that the information-based Entropy approach can systematically break down in the presence of outliers, while IQR-based robustness consistently maintains decision quality.

On the other hand, the width of the optimal β range that satisfies the advantages and stability conditions shows that the method can work without being confined to a narrow optimization window, i.e., it offers a wide range of applications to decision-makers. This structure, integrated with ranking methods such as VIKOR, is consistent with decision rules and also stands out in terms of ranking stability measured by SRD scores.

This demonstrates that IQRBOW-E not only achieves a low Q value but also possesses a structure that enhances the repeatability and reliability of decisions. Therefore, IQRBOW-E can be regarded not only as a methodological improvement but also as a tool that enhances confidence in the reliability and integrity of decision-making processes.

## 6. Conclusions

This study focuses on the structural nature of weighting in decision-making processes and proposes IQRBOW-E, a parametrically adjustable hybrid method that balances information sensitivity and robustness. Comprehensive experimental analyses conducted under various scenarios demonstrate that this method not only achieves high performance in average success measures but also in ensuring decision rules and ranking stability.

The adaptability of IQRBOW-E to data structures and its flexible β parameter are the most fundamental features that distinguish the method from fixed-structure classical approaches. The results of this study demonstrate that a scalable and modular weighting method capable of responding to the dynamic needs of decision support systems is feasible.

In this context, IQRBOW-E has the potential to offer reliable, adaptable, and interpretable solutions not only within the MCDM framework but also in more complex, multi-dimensional decision environments characterized by uncertainty and data irregularities. By enabling a tunable balance between robustness and information sensitivity through the β parameter, the method allows decision-makers to adjust weighting strategies dynamically based on the characteristics of the data. Future research could build on this flexibility by integrating IQRBOW-E with fuzzy logic, interval-based representations, or hierarchical decision models. Additionally, comparative analyses involving real-world data sets and alternative MCDM methods such as TOPSIS, MARCOS, or EDAS would further enrich the applicability and generalizability of the proposed approach.

This study operates under certain assumptions and within specific limitations that should be acknowledged. The simulation-based evaluation was conducted using synthetically generated decision matrices with normally distributed values and assumed independence among criteria. While this design provides a controlled environment for testing robustness, it may not fully capture the complexities of real-world decision problems. Moreover, the current analysis focused solely on quantitative performance indicators—such as average Q values, SRD scores, and decision stability—without addressing computational cost or decision-making efficiency. These limitations present valuable directions for future work, particularly for studies seeking to validate the method using empirical data or assess its performance across a broader spectrum of decision-making contexts.

## Figures and Tables

**Table 1:** Descriptive information about the scenarios.

Scenario	Number of Criteria	Number of Alternatives	Outlier Ratio	Objective
Sc1	10	10	0%	Basic performance assessment
Sc2	10	10	1%	Outlier data
Sc3	10	10	5%	Outlier data
Sc4	10	10	10%	Outlier data
Sc5	10	15	0%	Data size increase
Sc6	10	20	0%	Data size increase
Sc7	15	10	0%	Data size increase
Sc8	20	10	0%	Data size increase
Sc9	20	20	0%	Data size increase
Sc10	20	20	10%	Outlier data + Data size increase

**Table 2:** Summary table of scenarios.

Scenario	Optimal β	Average Q	Advantage (%)	Stability (%)	Min SRD	Friedman *p*
Sc1	0.425	0.0747	100	100	1.634	<0.001
Sc2	1	0.0496	100	100	1.195	<0.001
Sc3	1	0.0496	100	100	1.195	<0.001
Sc4	1	0.1136	0	100	1.634	<0.001
Sc5	0.8	0.0281	100	100	1	<0.001
Sc6	0.675	0.0124	100	100	1.22	<0.001
Sc7	0.95	0.0018	100	100	1	<0.001
Sc8	1	0	100	100	1	<0.001
Sc9	0.9	0.2571	100	100	1.268	<0.001
Sc10	0.975	0.3638	100	100	2.902	<0.001

## Data Availability

The raw data supporting the conclusions of this article will be made available by the authors upon request.
